# Potential impact of ezetimibe on patients with NAFLD/NASH: a meta-analysis of randomized controlled trials

**DOI:** 10.3389/fendo.2024.1468476

**Published:** 2024-10-08

**Authors:** BoLun Jiao, Bing Wang, BoYan Liu, Jin Zhao, YunHao Zhang

**Affiliations:** Department of Endocrinology, Jinzhou Central Hospital, Jinzhou, Liaoning, China

**Keywords:** meta-analysis, NAFLD, NASH, ezetimibe, randomized controlled trials

## Abstract

**Background:**

Non-alcoholic fatty liver disease (NAFLD) is now the most common cause of chronic liver disease. Studies have found that ezetimibe may be utilized as a supplemental treatment for NAFLD. Additionally, many clinical trials reported the potential impacts of ezetimibe on patients with NAFLD, although some conclusions remain controversial. Therefore, this study aimed to evaluate the effects of ezetimibe on patients with NAFLD.

**Method:**

Online search was conducted across databases including PubMed, Embase, Scopus, Web of Science, Cochrane Library, Wanfang, VIP, and CNKI to retrieve all relevant controlled studies on the treatment of NAFLD with ezetimibe from the inception of the databases until April 2024. This meta-analysis comprised 10 randomized controlled trials (RCTs). Statistical analysis was conducted using the Meta package in R v4.3.2.

**Results:**

A total of ten RCTs were included in this study, encompassing 578 patients (290 in the ezetimibe group and 288 in the control group) diagnosed with NAFLD/non-alcoholic steatohepatitis (NASH). The results indicated that ezetimibe significantly reduced levels of aspartate aminotransferase (*P* < 0.01), glutamyl transferase (γ-GT) (*P* < 0.01), total cholesterol (*P* < 0.01), low-density lipoprotein cholesterol (*P* < 0.01), high-sensitivity C-reactive protein (*P* < 0.01), and interleukin-6 (*P* < 0.01), and markedly increased levels of glycated hemoglobin (*P* = 0.02).

**Conclusions:**

Ezetimibe may partially improve transaminase levels and positively impact liver function in patients with NAFLD/NASH.

**Systematic Review Registration:**

https://www.crd.york.ac.uk/PROSPERO/, identifier CRD42023461467.

## 1 Introduction

Non-alcoholic fatty liver disease (NAFLD) refers to a set of chronic metabolic stress-induced liver damage caused by overnutrition and insulin resistance (IR) in genetically vulnerable populations (1, 2). The prevalence of NAFLD worldwide is estimated to be 30.05%, according to a meta-analysis on studies published between 1990 and 2019 (3). In addition, previous studies concluded that 11% of patients with NASH may develop cirrhosis within 15 years, and 13% of patients with NASH and cirrhosis may develop hepatocellular carcinoma (HCC) (4).

It was found that NAFLD was closely associated with glucose and lipid metabolism disorders, for instance, IR, obesity, fasting hyperglycosemia, dyslipidemia, altered adipokine profiles and other metabolic abnormalities (5–7). Among them, glucose metabolism disorders were considered to be the most closely related to NAFLD (8). Moreover, studies indicated that patients with diabetes mellitus (DM) are three times more likely to develop NAFLD than patients without DM (non-DM) (9). Similarly, the risk of NAFLD was elevated in patients with impaired glycemic index, such as fasting blood glucose (FBS), fasting insulin (FI), and homeostasis model assessment of insulin resistance (HOMA-IR) (9–11). In addition, cytokines have been implicated as key mediators of inflammation, fibrosis, and cirrhosis in NAFLD (12). Factors reported to be involved in the development and progression of NAFLD include interleukin-1β (IL-1β), interleukin-6 (IL-6), tumor necrosis factor-α (TNF-α), and C-reactive protein (CRP) (13–15).

Ezetimibe, a Niemann-Pick C1-Like 1 (NPC1L1) inhibitor, reduces lipid levels by blocking the absorption of cholesterol by the brush border of the small intestine (16). According to many studies, ezetimibe has been intensively investigated in animal models related to NAFLD. By boosting cholesterol efflux transporters and lowering levels of triglycerides (TG) and total cholesterol (TC), it can lessen hepatic lipid accumulation and fibrosis (17–20). Moreover, a study by Dong Yun Kim indicated that these effects were observed in both NAFLD and NASH animal models (21). Collectively, these findings lay the foundation for the clinical practice of ezetimibe.

Nevertheless, the clinical efficacy of ezetimibe remains controversial. Randomized controlled trials (RCTs) by Takeshita Y et al. and Park et al. demonstrated that ezetimibe significantly reduced serum TC levels and improved liver fibrosis scores in patients with NAFLD (22, 23). Conversely, studies by Yongin Cho et al., Loomba et al. and Davide Noto et al. discovered that ezetimibe was not associated with the significant improvements of hepatic steatosis or fibrosis in NAFLD (24–26). Additionally, ezetimibe is being studied in a wide range of NAFLD populations, with potential effects from different combination therapies (27, 28), varied treatment durations (22, 26), and different age groups (22, 29). Thus, the number of studies on potential effects of these factors is limited.

Therefore, to clarify the potential therapeutic effect of ezetimibe on NAFLD, a meta-analysis of RCTs retrieved from the database was carried out to collect biochemical, imaging, and histological measurement results, to comprehensively evaluate the efficacy of ezetimibe in NAFLD and offer valuable insights for future research in this domain.

## 2 Methodology

This study adhered to the PRISMA statement (30) and the study protocol was registered on October 2, 2023, on the PROSPERO (ID: CRD42023461467).

### 2.1 Literature search and data sources

#### 2.1.1 Search strategy

A systemic retrieval was carried out across eight databases: PubMed, Embase, Scopus, Cochrane Library, Web of Science, Wanfang, CNKI, and VIP. The search covered the period from the inception of each database until April 2024. Terms indexed in the MeSH database were used to retrieve articles, including: “Nonalcoholic Fatty Liver Disease”, “nonalcoholic steatosis hepatitis”, “NASH”, “NAFLD”, “ezetimibe”, “zetia”, “ezetrol”, “inegy”, “vytorin”, and other synonyms or keywords as part of the search strategy. Additionally, manual searches were conducted to identify crucial reviews and references cited within the included literature.

#### 2.1.2 Inclusion/exclusion criteria

The “PICOS” principle was followed to determine the inclusion criteria. Studies were included when the following criteria were met: (1) RCTs; (2) subjects aged 18 years and above with a clear diagnosis of NAFLD or NASH; (3) intervention: ezetimibe as monotherapy or in combination with placebo or other conventional treatment regimens; (4) outcomes assessing at least one of the following: biochemical indicators (AST, ALT, GGT, TC, TG, LDL, HDL, HbA1c, FPG, HOMA-IR, hs-CRP, IL-6), histological indicators related to the liver (NAS score, steatosis, ballooning), liver stiffness measurement (LSM), MR elastography (MRE), magnetic resonance imaging proton density fat fraction (MRI-PDFF) before and after treatment. Studies were excluded if they: (1) did not meet the inclusion criteria; (2) included pregnant or lactating participant, or in lactation. Two authors independently reviewed the relevant studies based on the eligibility criteria. Disagreements were arbitrated by a third author.

#### 2.1.3 Data extraction and assessment of quality

Two researchers autonomously extracted data and assessed the quality of included RCTs, with discrepancies resolved through discussion. For each eligible study, pertinent information was extracted utilizing a pre-structured data sheet, encompassing the first author’s last name, publication year, country of origin, sample size, gender distribution, body mass index (BMI), average age, intervention duration, interventions, and study outcomes. Bias risks were rated independently by two authors with the help of the assessment tool provided in Revman v5.4, to evaluate the generation of random sequences, allocation concealment, blinding of participants and personnel, blinding of outcome assessment, incomplete outcome data, and selective reporting. Items in the tool were categorized as “low”, “high”, or “unclear” risk of bias (28).

#### 2.1.4 Data statistics and analysis

In the study, all analyzed variables were continuous and presented as mean (Mean) ± standard deviation (SD). Statistical analysis of continuous outcome variables was conducted using R v4.3.2. The mean difference (MD) was utilized when outcome measures were in the same unit, whereas the standardized mean difference (SMD) was employed when measurement units or methodologies varied. A 95% confidence interval (CI) was calculated along with the synthesized effect size. Heterogeneity in these studies was quantitatively assessed by *I* ^2^. When heterogeneity was substantial for a synthesized outcome measure (*I* ^2^ > 50% and *P* < 0.05), a random-effects model was utilized, or a fixed-effects model was adopted. For studies exhibiting high heterogeneity (*I* ^2^ > 50%), subgroup analysis was performed based on variables such as age of subjects (≤ 50 years, > 50 years), duration of ezetimibe intervention (≤ 24 weeks, > 24 weeks), and the background regimen of the ezetimibe intervention group (regular diet and lifestyle under control, combination with statins, low-calorie low-fat diet for weight reduction, combination with polyene phosphatidylcholine capsules). These subgroup analyses were carried out to identify the potential heterogeneity sources among these studies. Sensitivity analysis was done for all synthesized outcomes using the random-effects model to determine the sources of heterogeneity and assess the stability of the results. Then, publication bias was assessed using Egger’s test when an outcome measure included 10 or more studies. Significant publication bias was confirmed when a *P* < 0.05.

## 3 Results

### 3.1 Literature search results and basic characteristics

**Figure 1:**
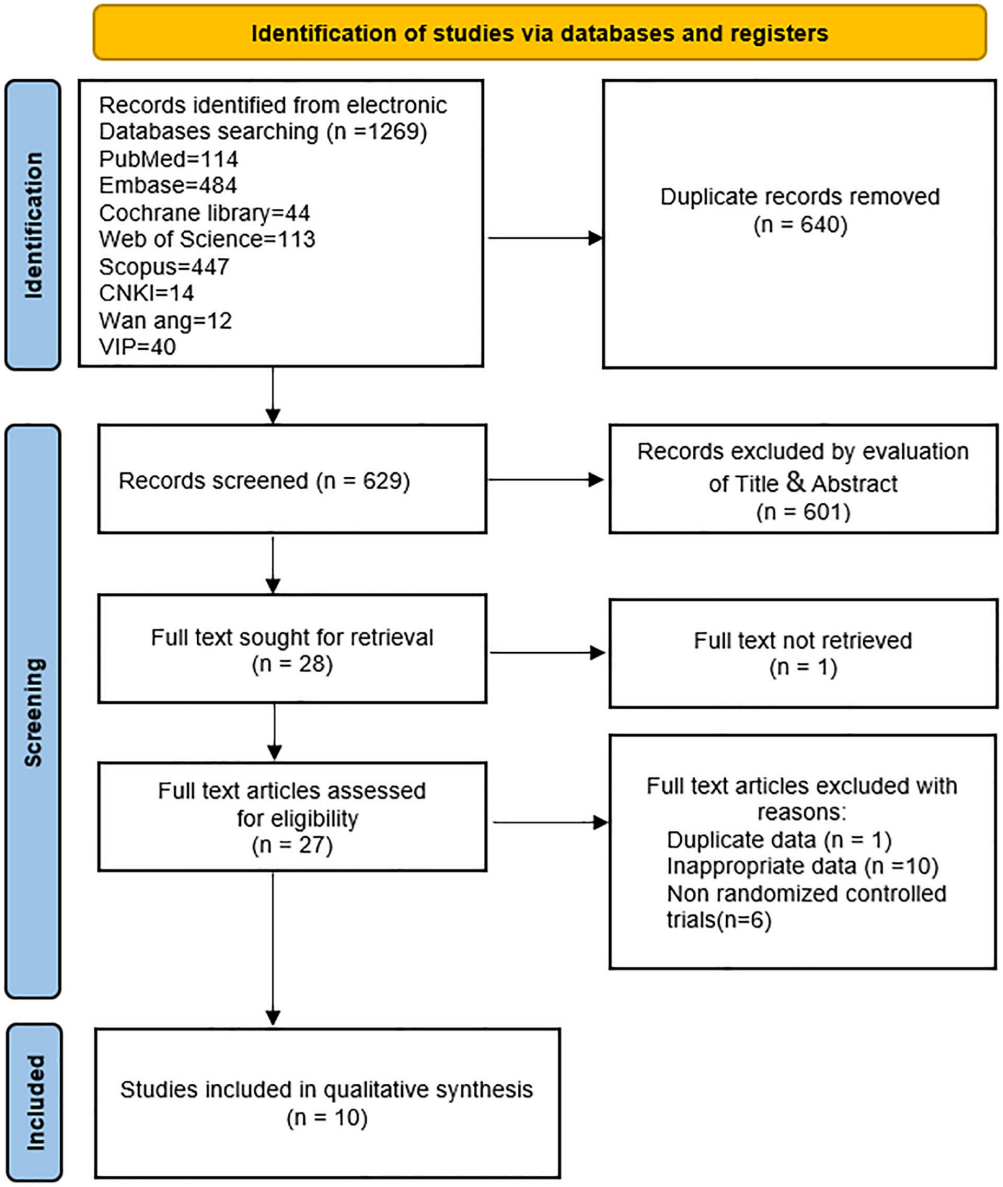
Flowchart of literature selection process.

Initially, this study retrieved 1,269 articles from databases, of which 640 were duplicates. Following the screening of titles and abstracts, 601 articles were excluded. Full texts of 28 articles were further assessed, resulting in the exclusion of 18 articles. Reasons for exclusion included inaccessible full text (one article) (31), data duplication (one article) (32), non-RCTs (six articles) (23, 33–37), and inappropriate data (10 articles) (38–47). Subsequently, 10 studies (22, 24–27, 29, 48–51) were incorporated into this meta-analysis. A comprehensive flowchart depicting the retrieval process is presented in **Figure 1**.

### 3.2 Basic characteristics and quality assessment

**Figure 2:**
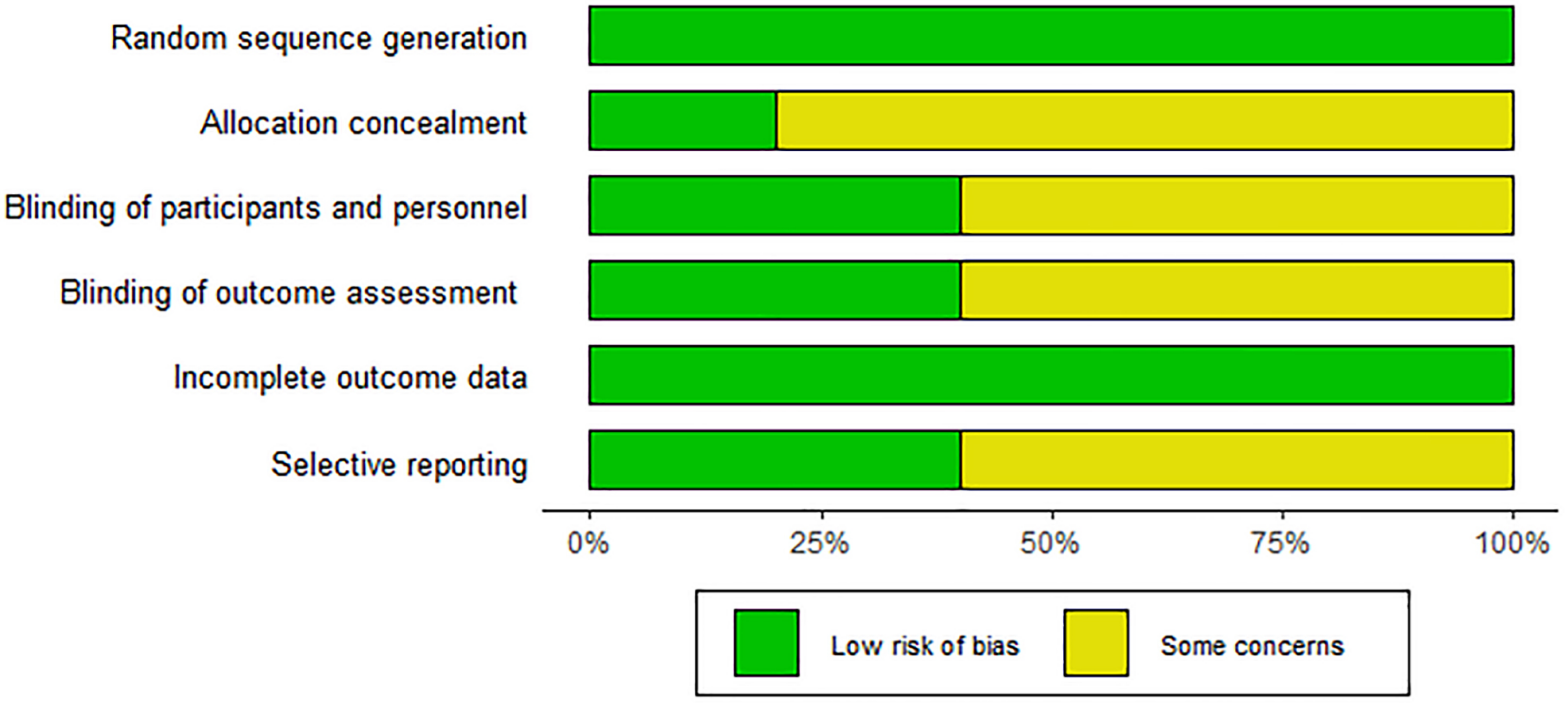
Risk of bias map for the included studies.

This study analyzed data from included articles covering a total of 578 subjects (290 received ezetimibe and 288 received controls). Characteristics of the 10 included studies are summarized in **Table 1**. The duration of intervention mentioned in the 10 studies ranged from 10 to 52 weeks. The intervention groups were all treated with ezetimibe 10 mg/d, and the control groups included a low-calorie diet + weight loss (2 studies), a lifestyle diet and basic treatment (3 studies), statin therapy (2 studies), polyene phosphatidylcholine capsules (2 studies), and placebo alone (1 study). Detailed information is presented in **Table 1**. The Cochrane Risk of Bias Assessment Tool was adopted to assess study biases. All 10 studies clearly defined their randomization methods; however, more than half were still considered to have some concerns regarding bias. Specifically, 8 studies did not provide detailed information on the generation and concealment of random allocation sequences. For the included studies, quality assessment results are presented in **Figure 2**.

**Table 1:** Inclusion of basic information for research.

Author (Year)	Country	Sample size (n)		Gender		BMI(kg/m2)	Average Age (years)	Intervention Time (Weeks)	NAFLD evaluation	Intervention		ALT(U/L)		AST(U/L)	
		Experimental	Control	Male	Female					Experimental	Control	Experimental	Control	Experimental	Control
Davide Noto 2022 (26)	Italy	20	20	NA	NA	28.2	NA	52	Biopsy	Ezetimibe 10mg + Low-Calorie Diet + Weight	Placebo + Low-Calorie Diet + Weight Loss	40.0 ± 7.8	31.0 ± 5.3	25.0 ± 3.5	24.0 ± 2.8
										Loss					
Yongin Cho 2022 (24)	Korea	31	33	40	30	28.5	51.4	24	Ultrasound	Ezetimibe 10mg + Rosuvastatin 5mg	Rosuvastatin 5mg	48.1 ± 26.6	69.7 ± 48.2	36.2 ± 17.0	49.3 ± 29.8
Ali Akbar 2013 (27)	Iran	29	33	35	27	30.3	40.6	10	Ultrasound	Ezetimibe 10mg + Diet and Lifestyle	Acarbose 100mg+ Diet and Lifestyle	94.6 ± 56.0	87.5 ± 27.8	54.7 ± 22.5	50.8 ± 16.6
DICK C 2010 (48)	Australia	15	10	15	10	33	57	16	MRI	Ezetimibe 10mg +Low-Calorie Diet + Weight Loss	Placebo + Low-Calorie Diet + Weight Loss	29.0 ± 7.74	32.0 ± 15.8	NA	NA
loomba2015 (25)	United States	25	25	19	31	33.4	49.3	24	Biopsy	Ezetimibe 10mg	Placebo	51.0 ± 7.3	47.0 ± 4.5	33.0 ± 5.8	32.0 ± 7.0
Yumie Takeshita 2014 (22)	Japan	17	14	20	31	29.2	52.7	24	Biopsy	Ezetimibe 10mg + Diet and Lifestyle	Diet and Lifestyle	53.2 ± 8.6	37.9 ± 6.8	41.8 ± 6.7	31.1 ± 4.4
Chen 2022 (49)	China	30	30	48	42	NA	43.5	24	Ultrasound	Ezetimibe 10mg + Diet and Lifestyle	Diet and Lifestyle	115.7 ± 28.4	113.2 ± 31.4	52.4 ± 19.6	53.4 ± 20.5
Li 2018 (29)	China	57	57	78	36	NA	46.5	24	Ultrasound	Ezetimibe 10mg + Atorvastatin 20mg	Atorvastatin 20mg	82.4 ± 15.5	82.7 ± 15.9	58.6 ± 11.7	58.4 ± 11.3
Xu 2017 (50)	China	38	38	45	31	NA	42.5	24	Ultrasound	Ezetimibe 10mg + Polyene Phosphatidyl choline 1368mg	Polyene Phosphatidyl choline 1368mg	114.7 ± 7.2	113.7 ± 8.1	85.9 ± 7.1	87.1 ± 6.4
Shen 2023 (51)	China	28	28	31	25	NA	43.8	48	Ultrasound	Ezetimibe 10mg + Polyene Phosphatidyl choline 1368mg	Polyene Phosphatidyl choline 1368mg	94.0 ± 13.8	92.5 ± 14.2	55.8 ± 11.3	58.7 ± 10.3

## 4 Outcome analysis

### 4.1 Biochemical indicators

#### 4.1.1 Liver function changes

**Figure 3:**
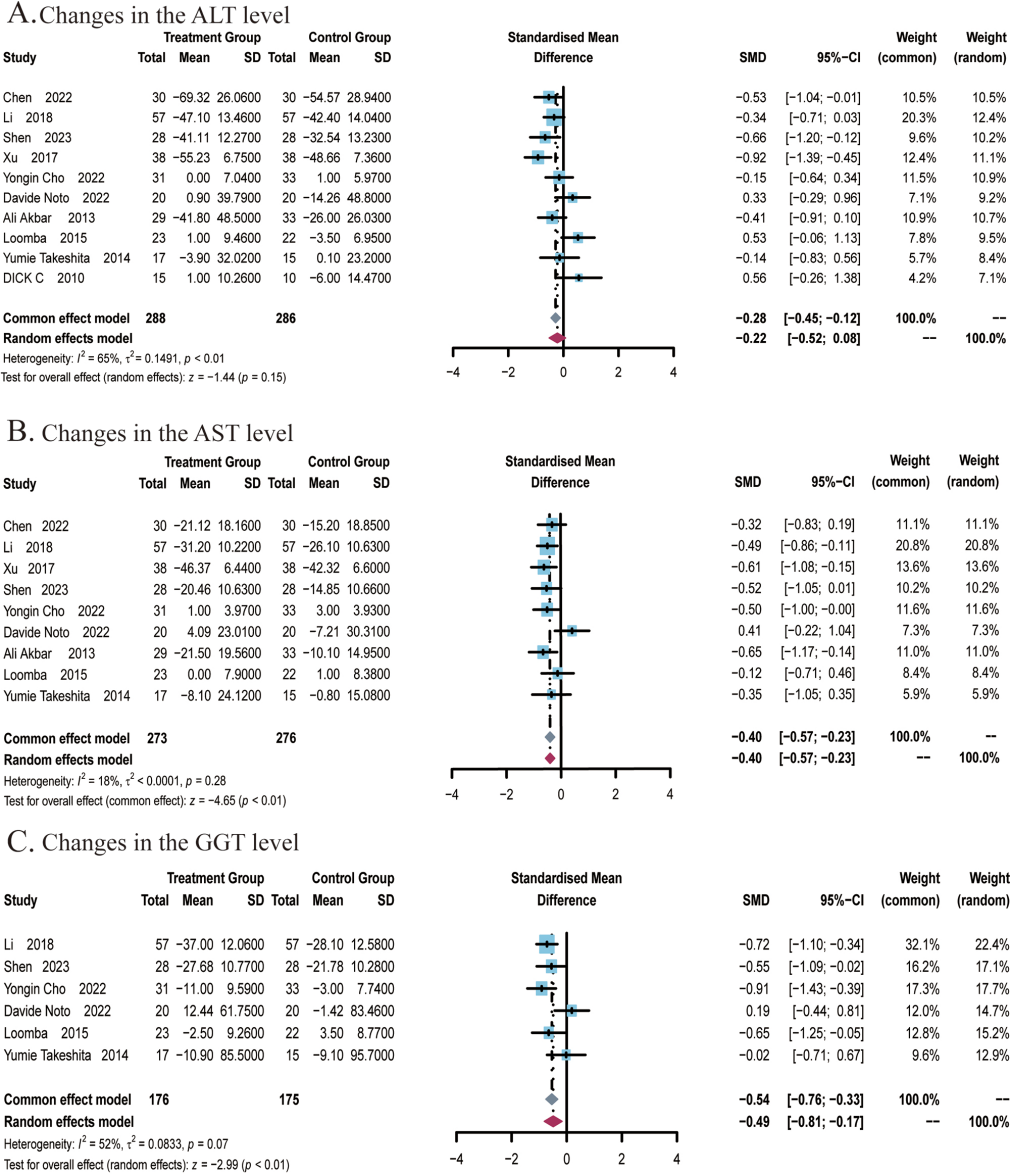
Meta-analysis of the impact of ezetimibe on liver function relative to the control group. **(A)** Changes in the AST levels. **(B)** Changes in the ALT levels. **(C)** Changes in the GGT levels.

A total of 10 studies (22, 24–27, 29, 48–51) covering 574 subjects reported the impact of ezetimibe on ALT levels. Heterogeneity was identified among these studies (*I* ^2^ = 65%; *P* < 0.01), therefore, a random-effects model was employed for analysis. The studies found no significant improvement in ALT levels in patients treated with ezetimibe relative to the control (SMD: -0.22; 95% CI: -0.52, 0.08; *P* = 0.15; **Figure 3A**). Subgroup analyses revealed significant reductions in ALT levels under the following conditions: when subjects were ≤50 years old (SMD: -0.40; 95% CI: -0.77, -0.04; *P* < 0.03), when ezetimibe was used alone in combination with regular diet and exercise (SMD: -0.40; 95% CI: -0.72, -0.08; *I* ^2^ = 0%; *P* = 0.01), and when combined with polyene phosphatidylcholine (SMD: -0.81; 95% CI: -1.16, -0.45; *I* ^2^ = 0%; *P* < 0.01) (**Supplementary Figures S1A, C**). Subgroup analysis based on the intervention duration did not yield significant changes in the results (**Supplementary Figure S1B**). Sensitivity analysis utilizing a random-effects model revealed that the exclusion of the study by Loomba et al. influenced the robustness of the results. Egger’s test indicated no significant publication bias (*P* = 0.0940).

A total of 9 studies (22, 24–27, 29, 49–51) covering 549 subjects reported the impact of ezetimibe on AST levels. No significant heterogeneity was noted among the studies (*I^2^* = 18%; *P* = 0.28), so a fixed-effects model was employed for analysis. The studies found that ezetimibe significantly reduced the AST level relative to the control (SMD: -0.40; 95% CI: -0.57, -0.23; *P* < 0.01; **Figure 3B**). The sensitivity analysis using a fixed-effects model indicated that no study significantly impacted the robustness of the synthesized results. Subgroup analyses indicated obvious reductions in AST levels when the intervention duration was ≤24 weeks (SMD: -0.46; 95% CI: -0.65, -0.27; *P* < 0.01), while age subgroups indicated no significant changes in the results (**Supplementary Figures S2A, B**). Egger’s test indicated no significant publication bias (*P* = 0.1340).A total of 6 studies (22, 24–26, 29, 51) covering 351 subjects reported the effect of ezetimibe on GGT levels. Among the studies, significant heterogeneity was observed (*I* ^2^ = 52%; *P* = 0.07), therefore, a random-effects model was employed. The studies found that ezetimibe significantly reduced GGT levels relative to the control (SMD: -0.49; 95% CI: -0.81, -0.17; *P* < 0.01; **Figure 3C**). The sensitivity analysis with a fixed-effects model suggested that no study significantly impacted the robustness of the synthesized results. Subgroup analyses found significant reductions in GGT levels when the intervention duration was ≤24 weeks (SMD: -0.66; 95% CI:-0.91, -0.40; *P* < 0.01) and when subjects were ≤50 years old (SMD: -0.66; 95% CI: -0.94, -0.39; *P* < 0.01) (**Supplementary Figures S3A, B**). Egger’s test revealed no significant publication bias (*P* = 0.1604).

#### 4.1.2 Lipid metabolism indicators

**Figure 4:**
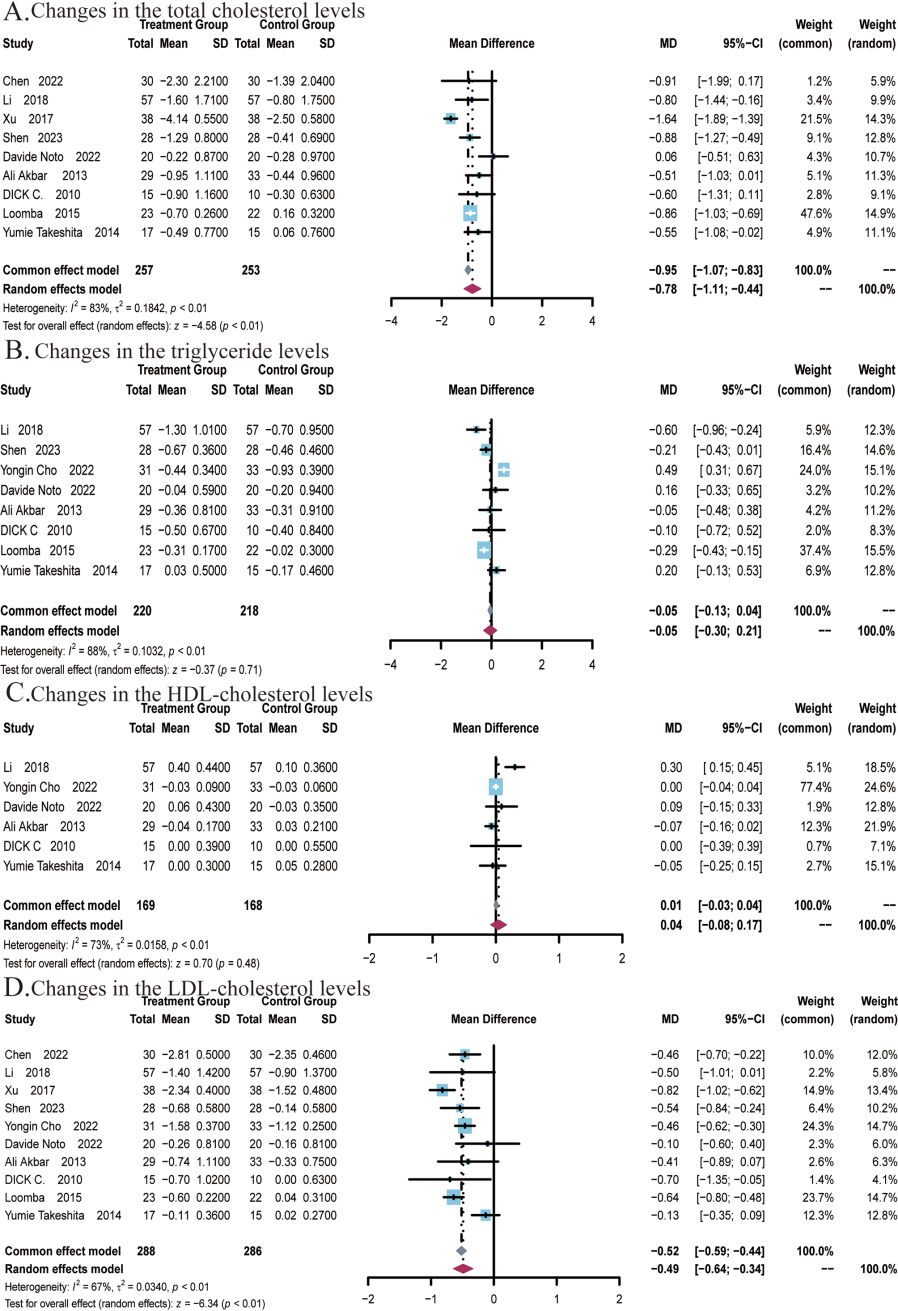
Meta-analysis of the impact of ezetimibe on lipid metabolism indicators relative to the control group. **(A)** Changes in the total cholesterol (TC) levels **(B)** Changes in the triglyceride (TG) levels. **(C)** Changes in the HDL cholesterol levels. **(D)** Changes in the LDL cholesterol levels.

A total of 9 studies (22, 25–27, 29, 48–51) covering 510 subjects reported the impact of ezetimibe on TC levels. Among these studies, significant heterogeneity was noted (*I* ^2^ = 83%; *P* < 0.01), therefore, a random-effects model was employed. The results suggested that ezetimibe treatment significantly reduced TC levels in patients with NAFLD relative to the control (MD: -0.78; 95% CI: -1.11, -0.44; *P* < 0.01; **Figure 4A**). Under the random-effects model, the sensitivity analysis revealed that no study significantly impacted the robustness of the synthesized results. Subgroup analysis suggested a significant reduction in TC levels when the intervention duration was ≤24 weeks (MD: -0.88; 95% CI: -1.23, -0.53; *P* < 0.01; **Supplementary Figure S4A**), while subgroup analysis regarding the average age of subjects indicated no significant changes in the results (**Supplementary Figure S4B**). Egger’s test for publication bias in the analysis of TC levels revealed no significant bias (*P* = 0.3055).

A total of 8 studies (22, 24–27, 48, 49, 51) covering 438 subjects reported the impact of ezetimibe on TG levels. Significant heterogeneity was present among the studies (*I* ^2^ = 88%; *P* < 0.01), thus, a random-effects model was employed. The studies found no significant improvement in TG levels in patients treated with ezetimibe relative to the control (MD: -0.05; 95% CI: -0.30, 0.21; *P* = 0.71; **Figure 4B**). Under the random-effects model, the sensitivity analysis suggested that no study significantly impacted the robustness of the synthesized results. Subgroup analyses regarding background medication and intervention duration revealed no significant changes in the results (**Supplementary Figures S5A, B**). However, significant reductions in TG levels were observed when the average age of subjects was ≤50 years (MD: -0.28; 95% CI: -0.39, -0.17; *P* < 0.01; **Supplementary Figure S5C**). Egger’s test for publication bias in the analysis of TG levels revealed no significant bias (*P* = 0.9770).

A total of 6 studies (22, 24, 26, 27, 29, 48) covering 337 subjects reported the impact of ezetimibe on HDL levels. The results did not exhibit significant differences across the ezetimibe and control groups. The heterogeneity in studies was identified as significant (*I^2^* = 73%; *P* < 0.01), and a random-effects model was adopted (MD: 0.04 mmol/L; 95% CI: -0.08, 0.17 mmol/L; *P* = 0.48; **Figure 4C**). According to the result, ezetimibe did not significantly improve HDL levels relative to the control. Under the random-effects model, the sensitivity analysis revealed that no study significantly impacted the robustness of the synthesized results. Subgroup analyses also did not yield significant changes in the results (**Supplementary Figures S6A, B**). Egger’s test for publication bias in the analysis of HDL levels revealed no significant bias (*P* = 0.5761).

A total of 10 studies (22, 24–27, 29, 48, 51) covering 574 subjects reported the impact of ezetimibe on LDL levels. The heterogeneity in studies was identified as significant (*I* ^2^ = 67%; *P* < 0.01), therefore, a random-effects model was utilized. The results indicated that ezetimibe treatment significantly reduced LDL levels in patients with NAFLD relative to the control (MD = -0.49 mmol/L, 95% CI: -0.64, -0.34 mmol/L, *P* < 0.01; **Figure 4D**). Under the random-effects model, the sensitivity analysis indicated that no study significantly impacted the robustness of the synthesized results. Subgroup analysis found that when the intervention duration was ≤24 weeks (MD: -0.46; 95% CI: -0.60, -0.32; *P* < 0.01), ezetimibe monotherapy combined with regular diet and lifestyle habits (MD: -0.31; 95% CI: -0.55, -0.07; *P* = 0.01), in combination with statins (MD: -0.46; 95% CI: -0.61, -0.31; *P* < 0.01), and combination with polyenyl phosphatidylcholine capsules (MD: -0.70; 95% CI: -0.97, -0.43; *P* < 0.01) all significantly reduced LDL levels (**Supplementary Figures S7A, B**). Subgroup analysis based on subjects’ ages did not yield significant alterations in the results (**Supplementary Figure S7C**). Additionally, Egger’s test for publication bias concerning the LDL level revealed no significant bias (*P* = 0.5677).

#### 4.1.3 Glycemic metabolism indicators

**Figure 5:**
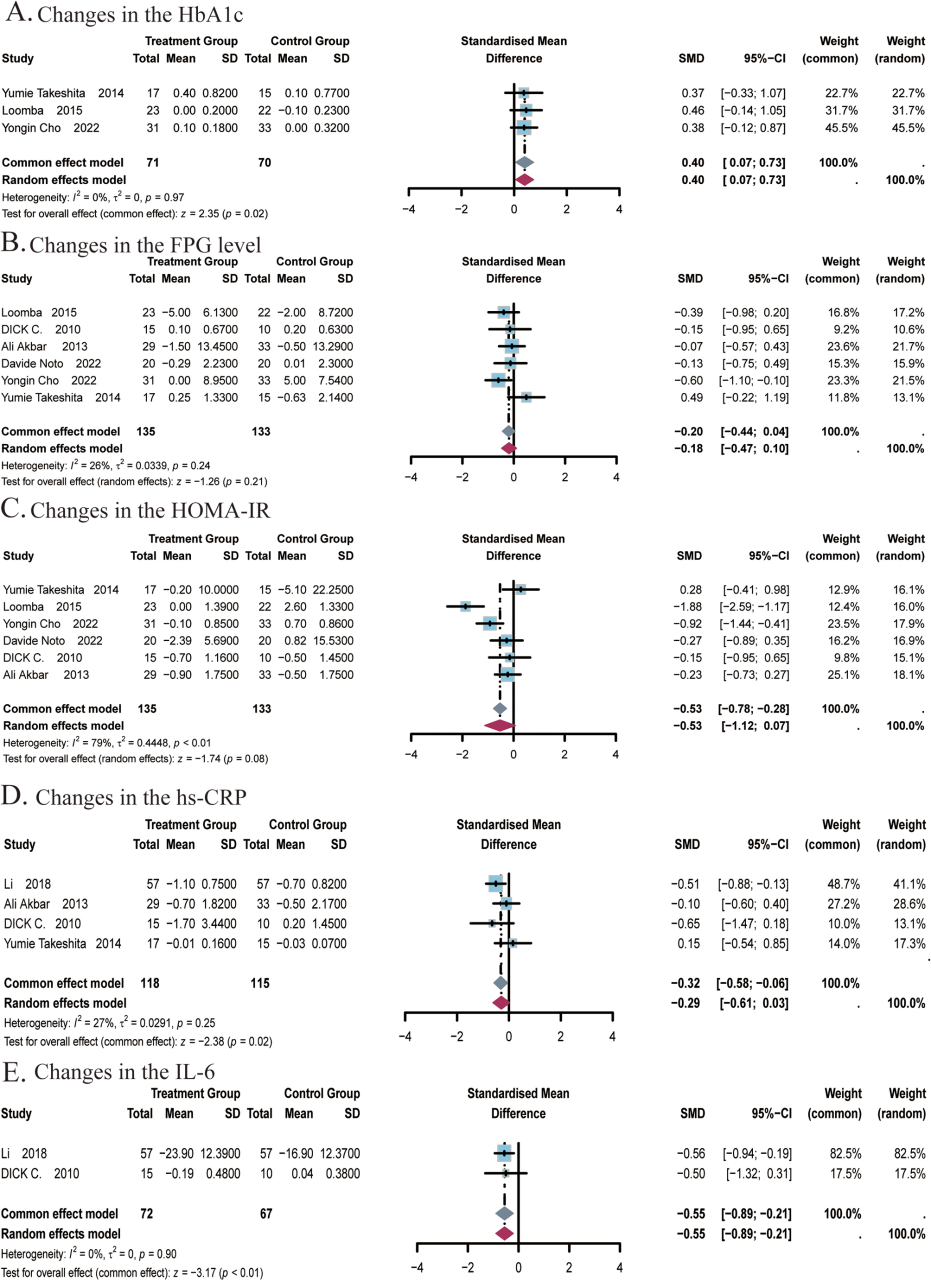
Meta-analysis of the impact of ezetimibe on glycemic indices and inflammatory markers relative to the control group. **(A)** Changes in the HbA1c. **(B)** Changes in the FPG level. **(C)** Changes in the HOMA-IR. **(D)** Changes in the hs-CRP. **(E)** Changes in the IL-6.

A total of 3 studies (22, 24, 25) covering 141 subjects reported the effect of ezetimibe on HbA1c levels. No significant heterogeneity was identified among the studies (*I^2^* = 0%; *P* = 0.97), so a fixed-effects model was adopted. Results suggested that ezetimibe treatment significantly increased HbA1c levels relative to the control (SMD: 0.40; 95% CI: 0.07, 0.73; *P* = 0.02; **Figure 5A**). Egger’s test identified no significant bias for this measure (*P* = 0.9597).

A total of 6 studies (22, 24–27, 48) covering 268 subjects reported the impact of ezetimibe on FPG levels and HOMA-IR. For FPG levels, no significant heterogeneity was observed among the studies (*I^2^* = 26%; *P* = 0.24), so a fixed-effects model was adopted. The analysis showed that ezetimibe treatment did not notably improve FPG levels in patients with NAFLD relative to the control (SMD: -0.20; 95% CI: -0.44, 0.04; *P* = 0.11; **Figure 5B**). For the analysis of HOMA-IR, significant heterogeneity was observed among the studies (*I^2^* = 79%; *P* < 0.01), leading to the selection of the random-effects model. Results suggested that ezetimibe treatment failed to significantly improve IR in patients with NAFLD relative to the control (SMD: -0.53; 95% CI: -1.12, 0.07; *P* = 0.08; **Figure 5C**). Sensitivity analysis utilizing a random-effects model revealed that the exclusion of the study by Yumie Takeshita et al. influenced the robustness of the results. Subgroup analysis based on the average age of subjects did not yield significant changes in the results (**Supplementary Figure S8A**). Egger’s tests for publication bias regarding the FPG level and HOMA-IR revealed no major bias, with respective *P* of 0.2960 and 0.9952.

#### 4.1.4 Inflammatory indicators

A total of 4 studies (22, 27, 29, 48) covering 233 subjects reported the impact of ezetimibe on hs-CRP levels. No notable heterogeneity was observed among the studies (*I^2^* = 27%, *P* = 0.25), allowing for analysis using a fixed-effect model. The results suggested that ezetimibe treatment significantly reduced hs-CRP levels in patients with NAFLD relative to the control (SMD = -0.32, 95% CI: -0.58, -0.06, *P* < 0.01; **Figure 5D**). Egger’s test for publication bias identified no significant bias (*P* = 0.6668).

A total of 2 studies (29, 48) covering 139 subjects reported the impact of ezetimibe on IL-6 levels. No remarkable heterogeneity was identified among the studies (*I^2^* = 0%, *P* = 0.90), allowing for analysis using the fixed-effect model. The results suggested that ezetimibe treatment significantly reduced IL-6 levels in patients with NAFLD relative to the control (SMD = -0.55, 95% CI: -0.89, -0.21, *P* < 0.01; **Figure 5E**).

#### 4.1.5 Hepatic steatosis and fibrosis

**Figure 6:**
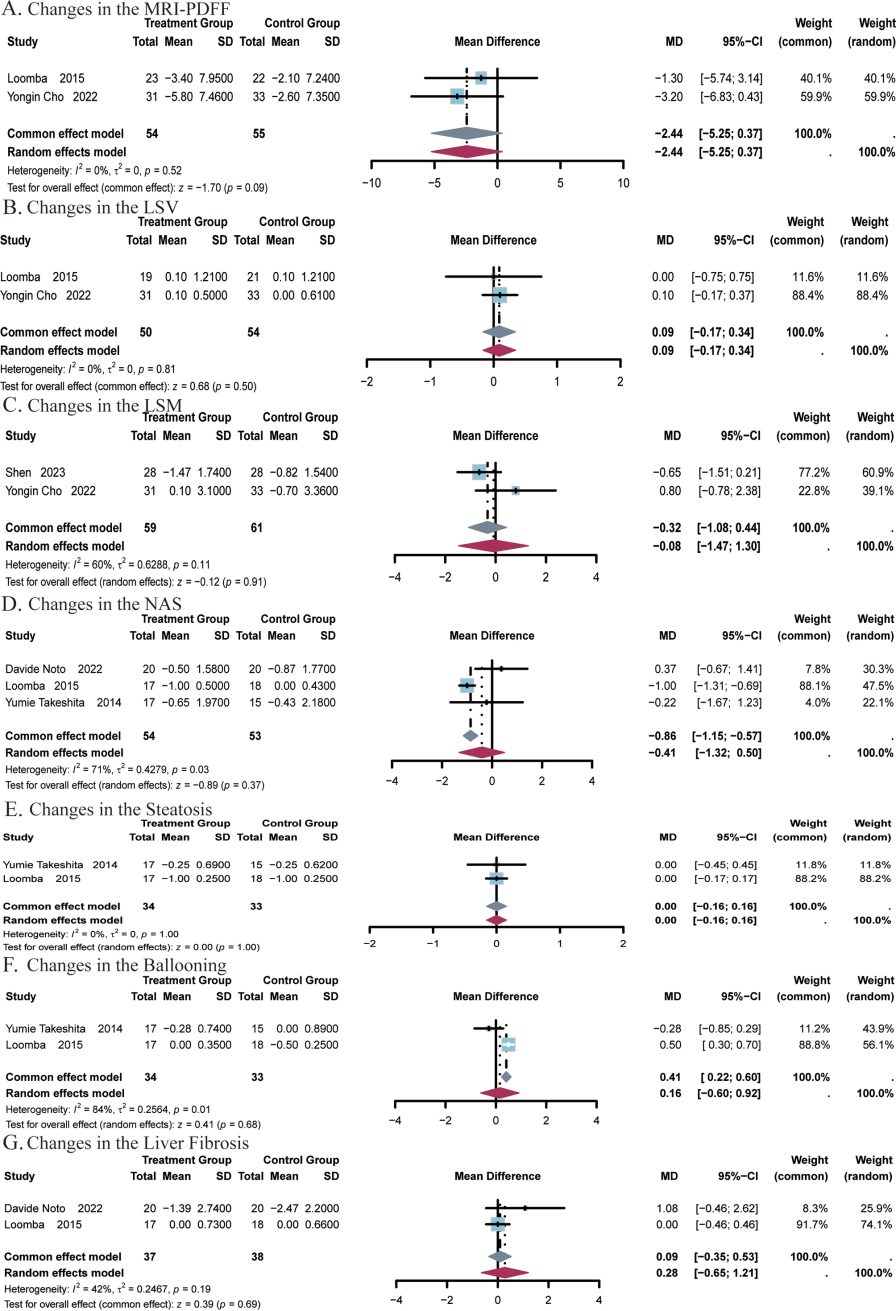
Meta-analysis of the impact of ezetimibe on hepatic steatosis and fibrosis relative to the control group. **(A)** Changes in the MRI-PDFF. **(B)** Changes in the LSV. **(C)** Changes in the LSM. **(D)** Changes in the NAS. **(E)** Changes in the Steatosis. **(F)** Changes in the Ballooning. **(G)** Changes in the Liver Fibrosis.

A total of 2 studies (24, 25) covering 109 subjects reported on the impact of ezetimibe on MRI-PDFF measurements. No significant heterogeneity was noted among the studies (*I^2^* = 0%, *P* = 0.52), and a fixed-effect model was applied. The results indicated no significant difference in MRI-PDFF measurements post-treatment with ezetimibe relative to the control (MD = -2.44, 95% CI: -5.25, 0.37, *P* = 0.09; **Figure 6A**). The effect of ezetimibe treatment on changes in LSV using 2D MRE was also reported in the above studies. No remarkable heterogeneity was observed among the studies (*I^2^* = 0%, *P* = 0.81), and a fixed-effect model was therefore adopted. The results indicated no significant difference in LSV post-treatment with ezetimibe relative to the control (MD = 0.09, 95% CI: -0.17, 0.34, *P* = 0.50; **Figure 6B**).

A total of 2 studies (24, 51) covering 120 subjects reported the impact of ezetimibe on LSM. Significant heterogeneity was identified among the two studies (*I^2^* = 60%, *P* = 0.11), and a random-effects model was therefore employed. The analysis showed no significant difference in LSM post-treatment with ezetimibe relative to the control (MD = -0.08, 95% CI: -1.08, 0.44, *P* = 0.91; **Figure 6C**).

A total of 3 studies (22, 25, 26) covering 107 subjects reported the impact of ezetimibe on NAS. Significant heterogeneity was present among the studies (*I^2^* = 71%, *P* = 0.03), and a random-effects model was therefore applied. The results indicated no significant difference in NAS post-treatment with ezetimibe relative to the control (MD = -0.41, 95% CI: -1.32, 0.50, *P* = 0.37; **Figure 6D**).

A total of 2 studies (22, 25) covering 67 subjects assessed the impact of treatment on steatosis and ballooning. No heterogeneity was identified for steatosis (*I^2^* = 0%, *P* = 1.00), so a fixed-effect model was used. For ballooning, significant heterogeneity was noted (*I^2^* = 84%, *P* = 0.01), and a random-effects model was applied. The results showed no significant improvement in either steatosis or ballooning post-treatment with ezetimibe relative to the control (steatosis: MD = 0.00, 95% CI: -0.16, 0.16, *P* = 1.00; ballooning: MD = 0.16, 95% CI: -0.60, 0.92, *P* = 0.68; **Figures 6E, F**). In addition, studies by Davide Noto et al., and Looma et al. (50, 51) covering a total of 75 subjects also evaluated the effects of liver fibrosis before and after treatment. No notable heterogeneity was observed among the studies (*I^2^* = 42%; *P* = 0.19), so a fixed-effects model was used for analysis. The results did not exhibit a significant difference in liver fibrosis post-treatment with ezetimibe relative to the control (MD = 0.09, 95% CI: -0.35, 0.53, *P* = 0.69, **Figure 6G**).

#### 4.1.6 Subgroup analysis

As heterogeneity was observed in some findings, subgroup analyses were conducted based on intervention duration, subject’s age, background medication and diagnostic evaluation of NAFLD in the experimental and control groups. Results showed that ezetimibe significantly improved certain liver metastasis enzymes and markedly reduced levels of AST and GGT in patients with NAFLD. Further subgroup analyses revealed, if the intervention duration was ≤ 24 weeks and the average age of subjects was ≤ 50 years, levels of AST and GGT also decreased substantially (**Supplementary Figures S1**, **S2**). Additionally, Park et al. reported that ezetimibe could improve ALT levels in patients with NAFLD (30), which yielded different results with this study. Subgroup analyses revealed that patients who aged ≤ 50 years or who had a diet high in polyunsaturated phosphatidylcholine may benefit more from ezetimibe. Moreover, major reductions were observed in both TG and TC levels among subjects aged ≤ 50 years, although the overall study did not demonstrate clear improvements in TG levels. In studies with an intervention duration of ≤ 24 weeks, remarkable reductions were observed in both TC and LDL-C levels. Furthermore, compared to monotherapy and adherence to routine diet and lifestyle practices, the combination of ezetimibe with statins and polyenyl phosphatidylcholine capsules significantly decreased LDL-C levels (**Supplementary Figures S4**-**S7**). Finally, we performed subgroup analyses of each indicator based on the different diagnostic criteria for patients with NAFLD. Results revealed there was significant heterogeneity in subgroups of liver enzyme indexes (ALT, AST, and GGT), and the heterogeneity within the group was greatly reduced. This implied that different diagnostic criteria for NAFLD may be one of the factors contributing to the observed heterogeneity. However, no significant changes were observed in the subgroup analyses of the other indicators (**Supplementary Figures S1**-**S8**).

## 5 Discussion

This meta-analysis comprised 10 RCTs to assess the effects of ezetimibe treatment on liver biochemical indicators, glucose and lipid metabolism indicators, steatosis, and fibrosis in patients with NAFLD. The findings of this study exhibited that ezetimibe could reduce levels of AST, GGT, TC, LDL-C, hs-CRP, and IL-6. However, no marked improvements were noted in levels of ALT, TG, HDL, FPG, HOMA-IR, steatosis, or fibrosis.

Yukiom et al. (52) conducted a systematic review to explore the effects of ezetimibe in the treatment of patients with NAFLD/NASH, and the results suggested that ezetimibe may impact liver steatosis, hepatocellular ballooning and ALT levels, which yielded different results with this study. Furthermore, ezetimibe could significantly lower the serum levels of TC, LDL-C, and inflammation-related markers, such as hs-CRP and IL-6. However, a study by Yukiom et al. did not carry out a thorough examination of markers linked to lipid metabolism and inflammation (52). These discrepancies may be mostly due to the fact the study by Yukiom et al. only included two RCTs. Thus, different designs of included trials may have reduced the validity of the evidence. As we only included RCTs in this study, this would no longer be a problem. Moreover, sufficient included RCTs allowed us to conduct more subgroup analyses.

The meta-analysis of this study demonstrated that, in comparison to the control group, the levels of TC and LDL-C in NAFLD patients treated with ezetimibe significantly decreased. Previous research has suggested that this effect may be attributed to the inhibition of ezetimibe on NPC1L1 (16). A meta-analysis involving 2,722 subjects receiving ezetimibe monotherapy, as reported by A. Pandor et al., revealed that ezetimibe significantly decreased patients’ cholesterol levels, aligning with the findings of this study (53). NPC1L1, serving as the molecular target of ezetimibe, has been implicated in promoting the uptake of cholesterol when overexpressed in cell studies (54, 55). Ezetimibe is recognized to have the capability to inhibit NPC1L1-dependent cholesterol uptake (56, 57). Research by Liang Ge et al. demonstrated that cholesterol specifically stimulates the internalization of NPC1L1, a process that necessitates actin and the clathrin/AP2 complex. The blockade of NPC1L1 endocytosis markedly diminishes cholesterol internalization, suggesting that NPC1L1 facilitates cholesterol uptake via its vesicular endocytic pathway (56, 58). Ezetimibe can impede the incorporation of NPC1L1 into clathrin-coated vesicles, consequently inhibiting cholesterol uptake (56). An animal experiment conducted by Garcia-Calvo M et al. revealed that isotopically labeled ezetimibe could bind to the brush border membrane vesicles of intestinal cells in wild-type mice, whereas this binding was absent in NPC1L1 knockout mice (54). Moreover, DNA sequence variations in NPC1L1 were found to correlate with enhanced response to ezetimibe treatment in research conducted by Jason S. Simon et al. (59).

Moreover, regarding glycemic control parameters, this study did not exhibit significant alterations in FPG levels and HOMA-IR following ezetimibe treatment. However, a significant increase in the HbA1c level was observed. Research by Erqou S et al. also suggested an increased risk of new-onset diabetes mellitus and elevated HbA1c levels associated with ezetimibe treatment. Therefore, from studies, conflicting evidence exists (60). A meta-analysis conducted by Huijin Wu et al., which encompassed 16 RCTs assessing patients with dyslipidemia or NAFLD with or without diabetes mellitus, indicated that HbA1c levels were not affected by ezetimibe (WMD: 0.07%, 95% CI: -0.07,0.20%, *P* = 0.627) (61). However, it is noteworthy that the majority of the population evaluated in the meta-analysis consisted of patients with or without diabetic obesity and dyslipidemia, with only one study including patients with NAFLD (20). Upon comparing all available data, our study has presented additional findings indicating that ezetimibe might elevate HbA1c levels in patients with NAFLD. Given the limited number of included studies and subjects included in this analysis, it is plausible that the results may be influenced by these factors. Currently, there are no reports detailing the precise impact of ezetimibe on HbA1c levels in patients with NAFLD. It is imperative to acknowledge that NAFLD frequently coexists with other metabolic-related conditions, such as metabolic syndrome and diabetes mellitus. As HbA1c serves as a pivotal indicator of these diseases, elucidating whether HbA1c levels are impacted by ezetimibe holds significant importance in evaluating disease outcomes. Therefore, heightened attention and further large-scale RCTs are needed to provide clarity on the matter.

Our study also identified that ezetimibe treatment exhibited a discernible anti-inflammatory effect, which is able to decrease levels of hs-CRP and IL-6. Research by Krysiak et al. demonstrated that ezetimibe monotherapy (10 mg/day) can markedly lower hs-CRP levels, with the extent of ezetimibe’s effect on hs-CRP correlating with the sensitivity of patients to insulin (62). For example, for individuals with normal insulin sensitivity, ezetimibe alone exhibited minimal anti-inflammatory effects. Moreover, this study revealed that the reduction in hs-CRP induced by ezetimibe alone and in combination with simvastatin was time-dependent and not contingent upon lipid levels (54). Research conducted by Arab S M et al. similarly suggested that the combination of ezetimibe with statins can markedly decrease hs-CRP levels (WMD: -0.2 mg/L; 95% CI: [-0.4, -0.1]; *P* < 0.001), with changes in hs-CRP levels significantly correlating with alterations in serum LDL-C (63). Other studies have also indicated that ezetimibe can inhibit the infiltration of inflammatory cells and the release of cytokines (62, 64). A study by Toshiyuki et al. in animals suggested that following the administration of a high-fat diet to medaka fish, the number of inflammatory cells deep within the liver tissue observed under high magnification at weeks 8 and 12 was more abundant than that in the control group. However, after administration with ezetimibe alongside a high-fat diet, there was no increase in inflammatory cells in the liver tissue, indicating that ezetimibe can exert an inhibitory effect on the infiltration of inflammatory cells (64). Additionally, another study has reported that ezetimibe can also decrease relatively high levels of IL-6 and IL-1β (65).

While previous studies have demonstrated that the histological resolution of NASH correlates with reductions in NAS scores and improvements in fibrosis levels (66, 67), this study did not observe clear histological outcomes or improvements in fibrosis. Similarly, although a meta-analysis conducted by Hyo Young Lee et al. demonstrated a reduction in NAS scores, no significant changes in hepatic steatosis were observed (68). In this study, only three studies included NAS scores as a secondary outcome measure (22, 25, 26). However, these three studies had small sample sizes and only a 6-month observation period. They focused on different racial demographics, potentially limiting the ability to draw conclusive findings due to these disparities. Additionally, two studies conducted by Loomba and Yongin Cho also assessed MRI-PDFF, MRE, and LSM. For these measures, but our meta-analysis results did not reveal the therapeutic effects of ezetimibe (24, 25). Among the included studies, the study by Loomba et al. found that ezetimibe monotherapy did not prominently reduce liver fat compared to placebo (25). Conversely, the study by Yongin Cho et al. suggested that compared to statin monotherapy, ezetimibe as a combination therapy might have a more significant effect in reducing liver fat (24). Thus, while the reduction of liver fat by ezetimibe remains unclear, the potential positive effects of ezetimibe as a combination therapy on liver fat are not negated by this uncertainty.

In this study, we performed subgroup analyses based on different diagnostic criteria for NAFLD. Results indicated that significant differences were observed in subgroups of liver enzyme indexes (ALT, AST, and GGT), but no substantial differences were discovered for the others. It should be pointed out that significant variations in different diagnostic procedures were only present in certain indicators, and we cannot yet assert that there is a great disparity in the efficacy of ezetimibe among populations diagnosed using different criteria.

Among the included studies, adverse events that occurred during the use of ezetimibe were only reported in a study carried out by Davide Noto et al. Compared with the control group, no significant increase in common serious adverse events (SAE) or treatment-emergent adverse events (TEAE) was observed following treatment with ezetimibe. In the ezetimibe group, one case was deemed to be an adverse event resulting from home injury, which was determined to be unrelated to the ezetimibe treatment (26).

It has been preliminarily confirmed that ezetimibe could improve liver enzyme levels, enhance the anti-inflammation effect, and lower serum levels of TC and LDL-C in patients with NAFLD/NASH. Compared with ezetimibe, novel drugs with the potential to treat NAFLD/NASH are also in the research and verification phases. For instance, GLP-1 receptor agonists can reduce body weight, improve IR, lower liver enzyme levels, and reduce liver fat content in patients with T2DM, which may be beneficial for the treatment of NAFLD/NASH (69). According to a Japanese study, subcutaneous administration of dulaglutide at a dose of 0.75 mg/week for 12 weeks could reduce body weight and improve liver enzyme levels in T2DM patients with NAFLD (70). Additionally, a study by Armstrong MJ et al. found that a subcutaneous administration of liraglutide at a dose of 1.8 mg/d in patients with NASH could improve hepatic histopathology, with regression and no fibrotic progression in NASH (71). However, the use of liraglutide is commonly linked to gastrointestinal adverse reactions such as diarrhea, constipation, and loss of appetite (71). Furthermore, SGLT2 inhibitors (SGLT2i) have been thought to significantly reduce transaminases and improve hepatic steatosis in current research (72). A meta-analysis revealed that the liver fat content, liver enzyme levels, BMI, and inflammatory markers were improved in Asian T2DM patients with NAFLD after being treated with SGLT2i (73). Nevertheless, adverse effects such as hypoglycemia, ketoacidosis, urinary tract infections and genital infections should also be noted when applying SGLT2i (74). Moreover, farnesoid X receptor (FXR) is a nuclear receptor activated by bile acids (BA) that are highly expressed in the liver and intestinal system, and has become a hotspot of research on NAFLD (75). Obeticholic acid (OCA) is one of the representative FXR agonists (76). It has been shown that FXR controls were associated with multiple pathogenic pathways, and its activation not only effectively inhibited the progression of NASH, but also reversed its consequences, especially liver fibrosis (77). According to FLINT and REGENERATE trials, remarkable improvements were noted in the fibrosis of patients with NASH after being treated with FXR ligands, but no substantial changes were observed in regression in NASH (78, 79). However, long-term OCA treatment was linked to diseases of the skin and subcutaneous tissue, gastrointestinal diseases and elevated cholesterol levels (80). That is to say, the OCA use in patients with NAFLD/NASH may be restricted by these adverse reactions. In conclusion, attention should be paid to the efficacy comparison of ezetimibe and other treatments for NAFLD/NASH in the future. Later research demonstrated that no studies have compared the efficacy of ezetimibe directly or indirectly with GLP-1 agonists, FXR, SGLT2 inhibitors and other medications. Thus, more large-scale RCTs are required to confirm and verify these conclusions. Meanwhile, it would be worthwhile to investigate the efficacy when these medications are combined under safe circumstances.

### 5.1 Advantages and limitations

This study is the first meta-analysis based on RCTs. Initially, data extraction and analyses were performed based on RCTs of ezetimibe, to explore the efficacy of ezetimibe in specific populations, and provide data support for the future clinical use of ezetimibe. However, our study has certain limitations. First, the limited number of included studies and small sample sizes of most RCTs potentially diminished the significance of the results. Second, the follow-up periods of the included studies were relatively short, which may not entirely reflect the true effects. Hence, more studies on its long-term efficacy are required for additional validation in the future. Third, most of the studies included in this article are from Asia, so the applicability of the results to non-Asian populations needs to be improved. Therefore, future research should employ unbiased methods for standardized, prospective, multicenter, long-term, and large-scale RCTs. Ultimately, according to the subgroup and sensitivity analyses, differences in background treatment may impact the ultimate outcomes. In terms of adverse drug events, only a single study addressed the adverse reactions of ezetimibe. It is anticipated that future researchers will prioritize adverse events and undertake more exhaustive and detailed investigations to assemble robust evidence.

## 6 Conclusion

This study showed that ezetimibe could partially reduce transaminase levels and provide certain benefits to liver function in patients with NAFLD or NASH. Additionally, ezetimibe significantly lowers serum TC and LDL-C levels in patients with NAFLD, and shows some signs of reducing inflammation. Furthermore, we found that ezetimibe might offer greater benefits in reducing liver transaminase, and serum levels of TC and LDL-C for patients aged ≤ 50 years and with an intervention period of ≤ 24 weeks. However, we did not observe any important effects of ezetimibe on liver steatosis or fibrosis. To some extent, the results of this study provide evidence-based support for the clinical use of ezetimibe in NAFLD/NASH, and lay the foundation for larger, better-designed RCTs in other populations in the future.

## Data Availability

The original contributions presented in the study are included in the article/**Supplementary Material**. Further inquiries can be directed to the corresponding author.
