# Supplementary material for: Potential impact of ezetimibe on patients with NAFLD/NASH: a meta-analysis of randomized controlled trials
Source: Front Endocrinol (Lausanne). 2024 Oct 8;15:1468476. doi: 10.3389/fendo.2024.1468476 (PMC11493694; doi:10.3389/fendo.2024.1468476)

**Fig S1.** Meta-analysis of randomized controlled trials evaluating the effect of application of ezetimibe on ALT levels in NAFLD patients different control groups, treatment backgrounds (a), intervention periods (b), ages (c)and NAFLD evaluation(d).


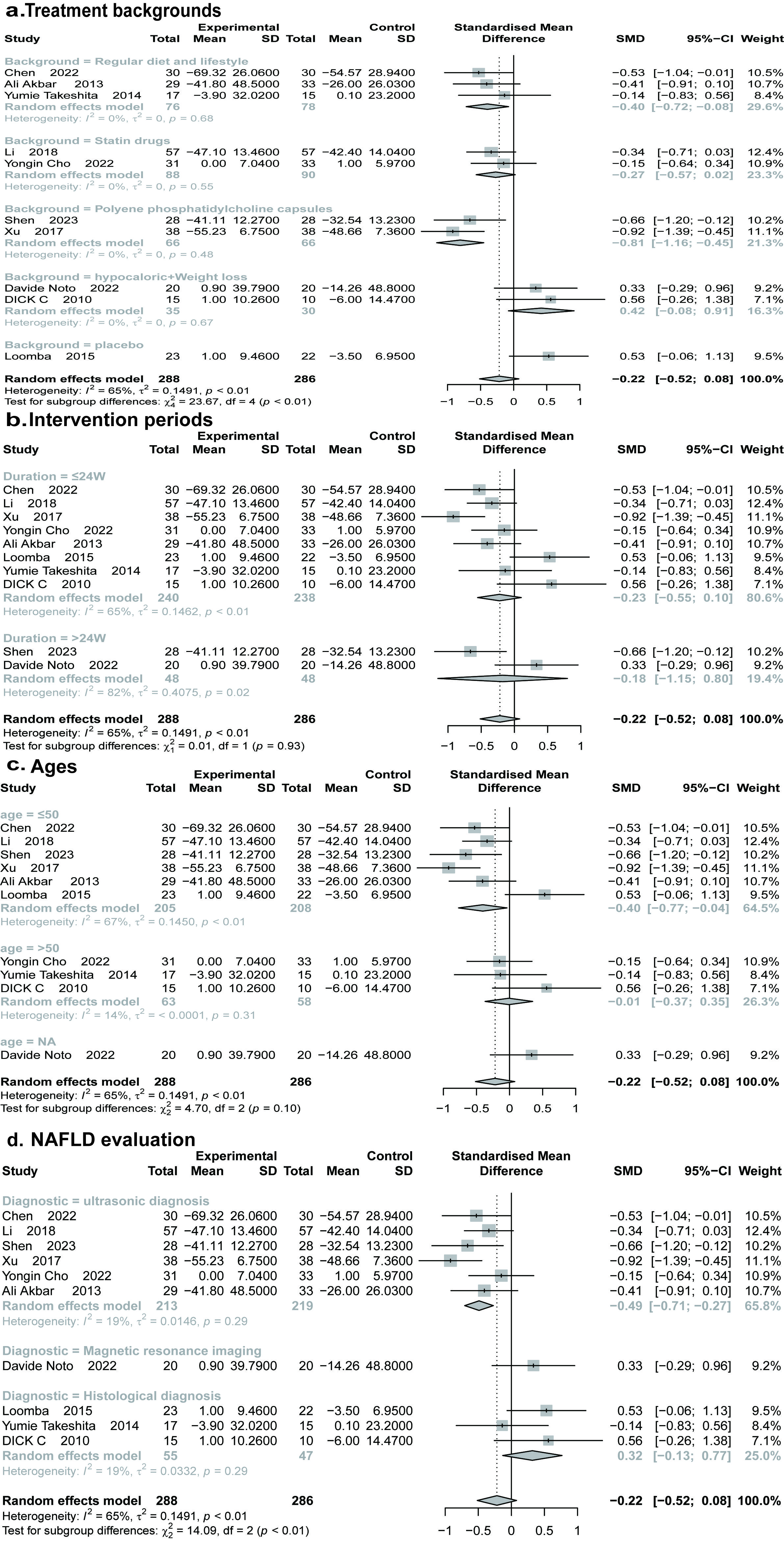


**Fig S2.** Meta-analysis of randomized controlled trials evaluating the impact of ezetimibe on AST levels in NAFLD patients across different intervention periods (a) , age groups (b) and NAFLD evaluation(c).


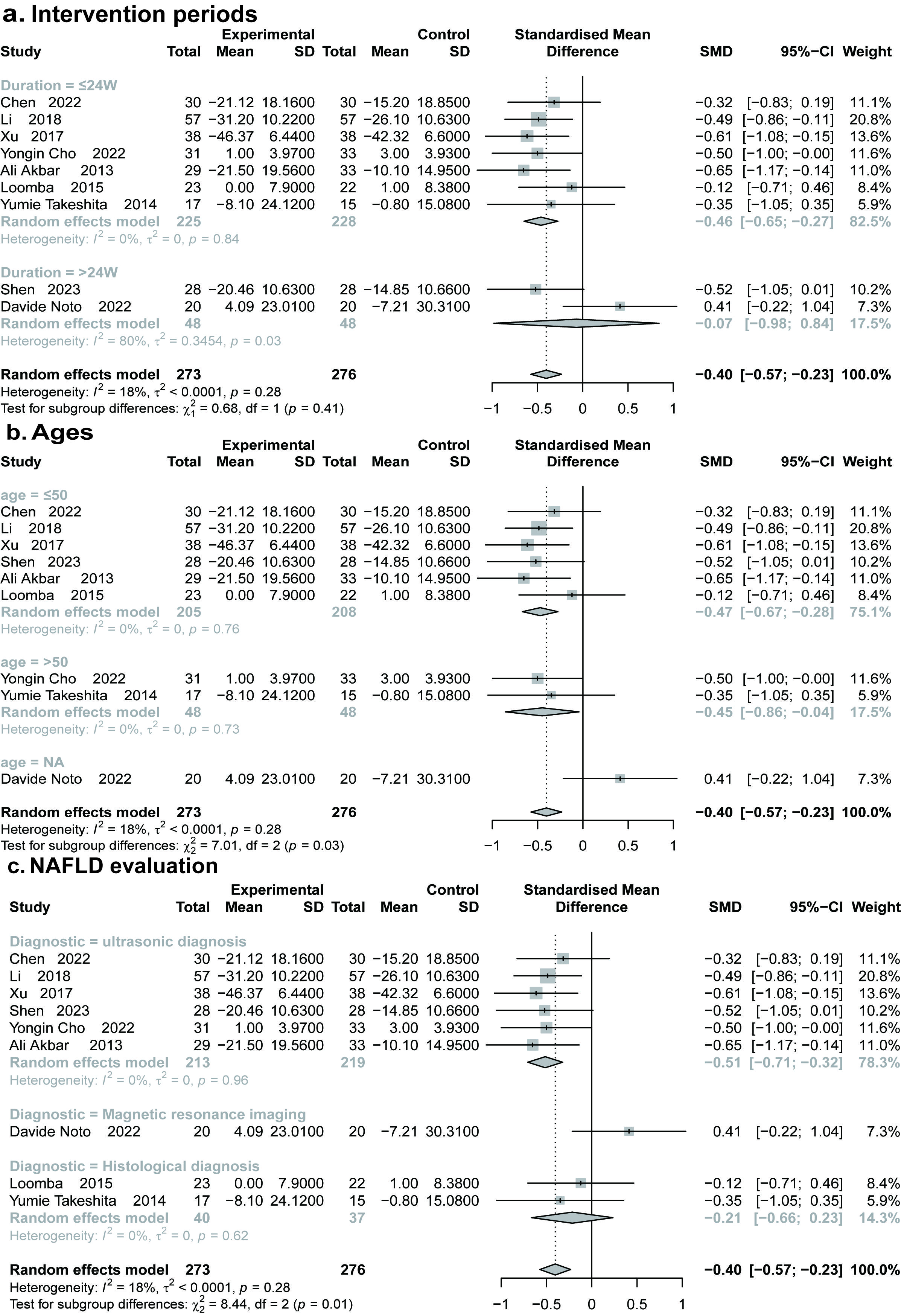


**Fig S3.** Meta-analysis of randomized controlled trials evaluating the impact of ezetimibe application on GGT levels in NAFLD patients across different intervention periods (a), ages (b) and NAFLD evaluation(c).


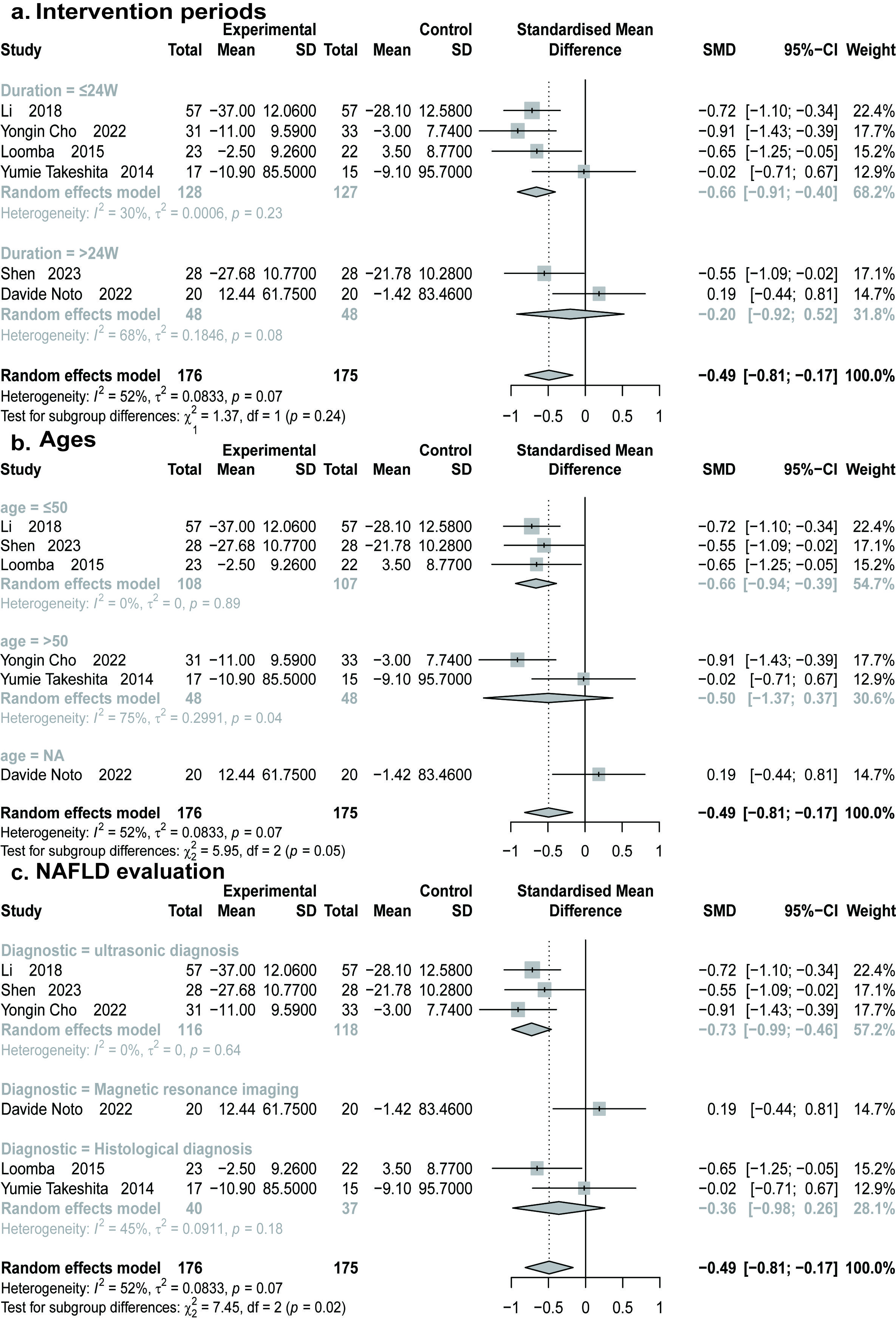


**Fig S4.** Meta-analysis of randomized controlled trials evaluating the impact of ezetimibe on TC levels in NAFLD patients across different intervention periods (a), age groups (b) and NAFLD evaluation(c).


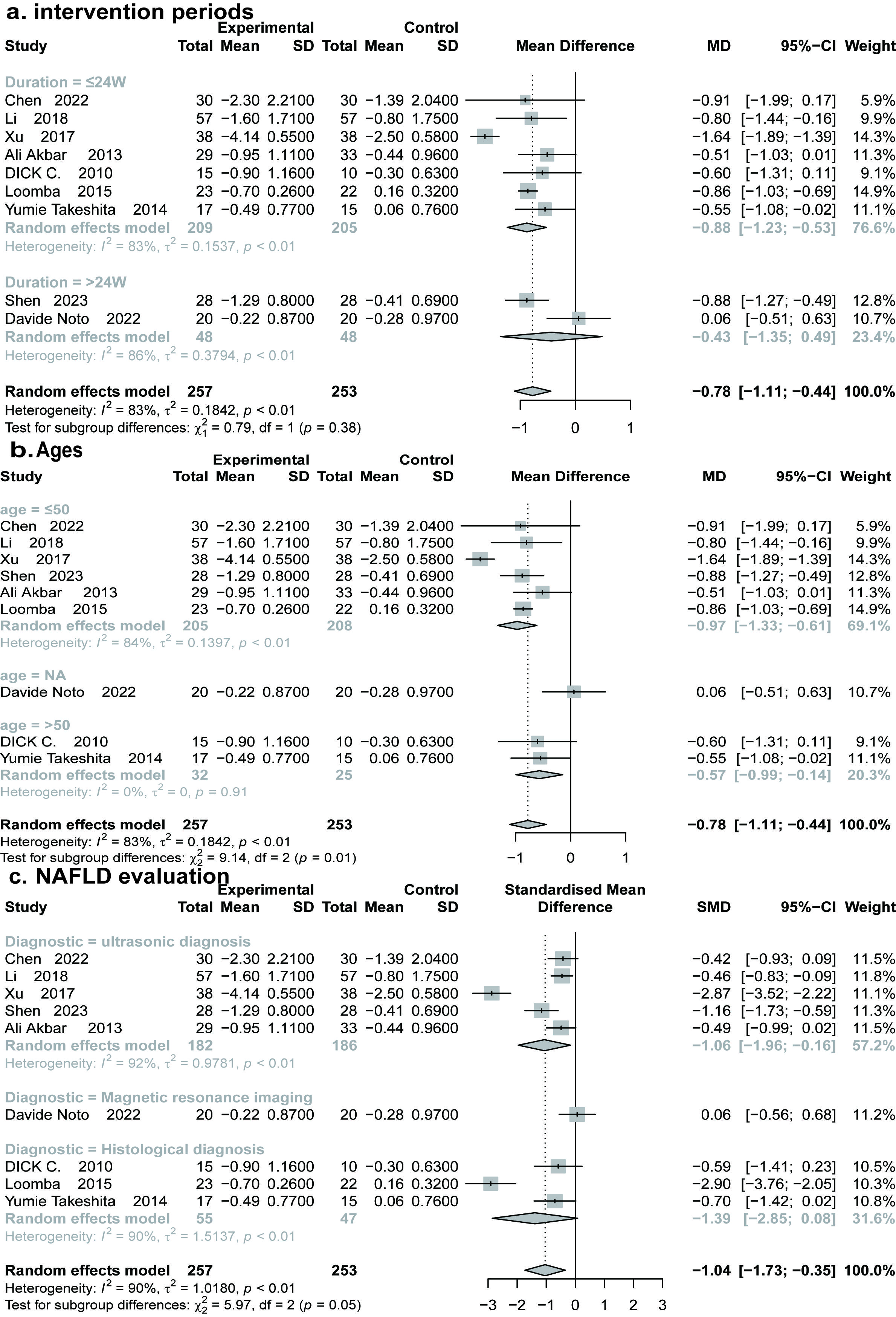


**Fig S5.** Meta-analysis of randomized controlled trials evaluating the impact of ezetimibe application on TG levels in NAFLD patients across different control groups, treatment backgrounds (a), intervention periods (b), ages (c) and NAFLD evaluation(d).


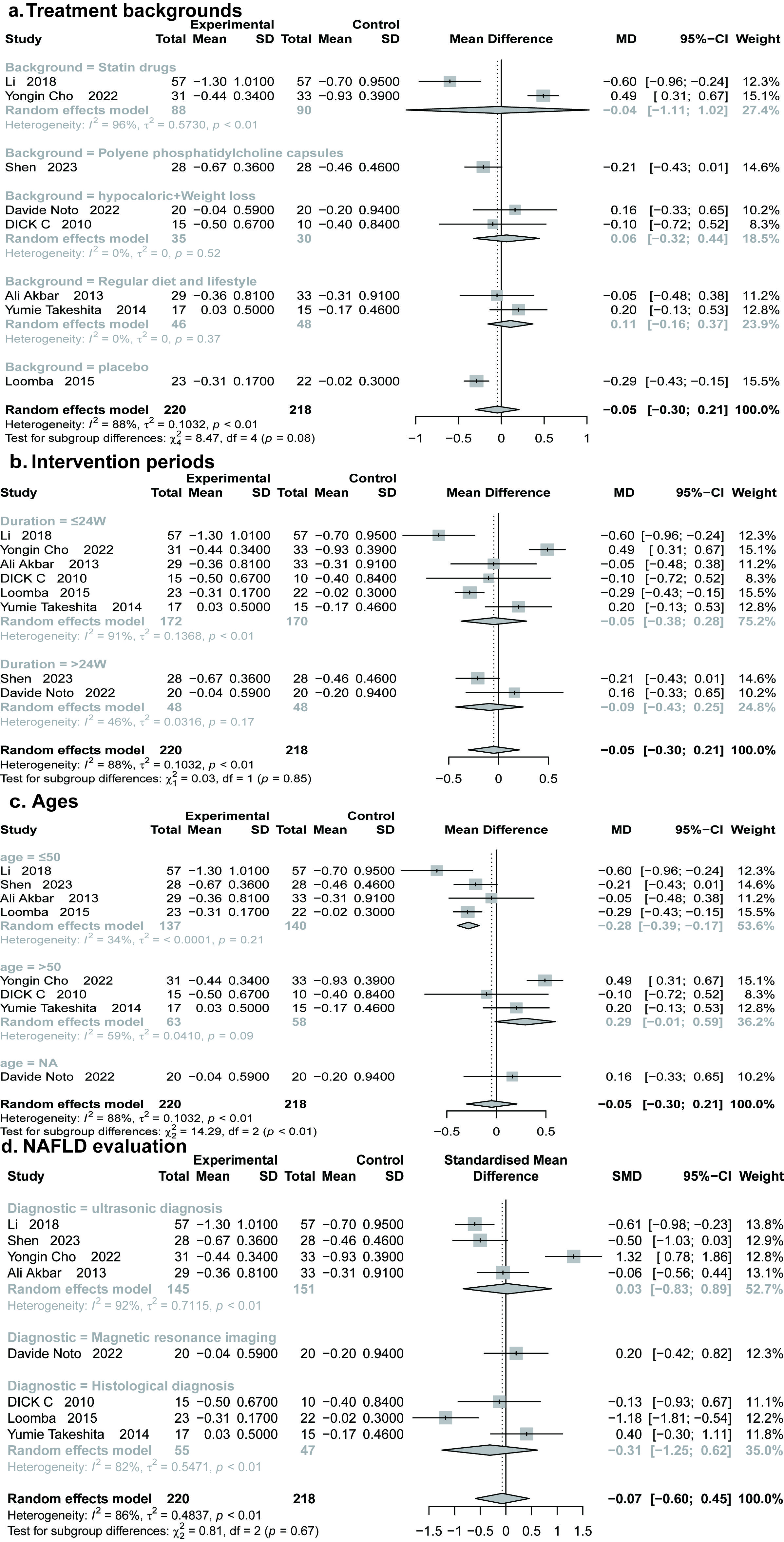


**Fig S6.** Meta-analysis of randomized controlled trials evaluating the impact of ezetimibe application on HDL-C levels in NAFLD patients across different control groups, ages (a), treatment backgrounds (b) and NAFLD evaluation(c).


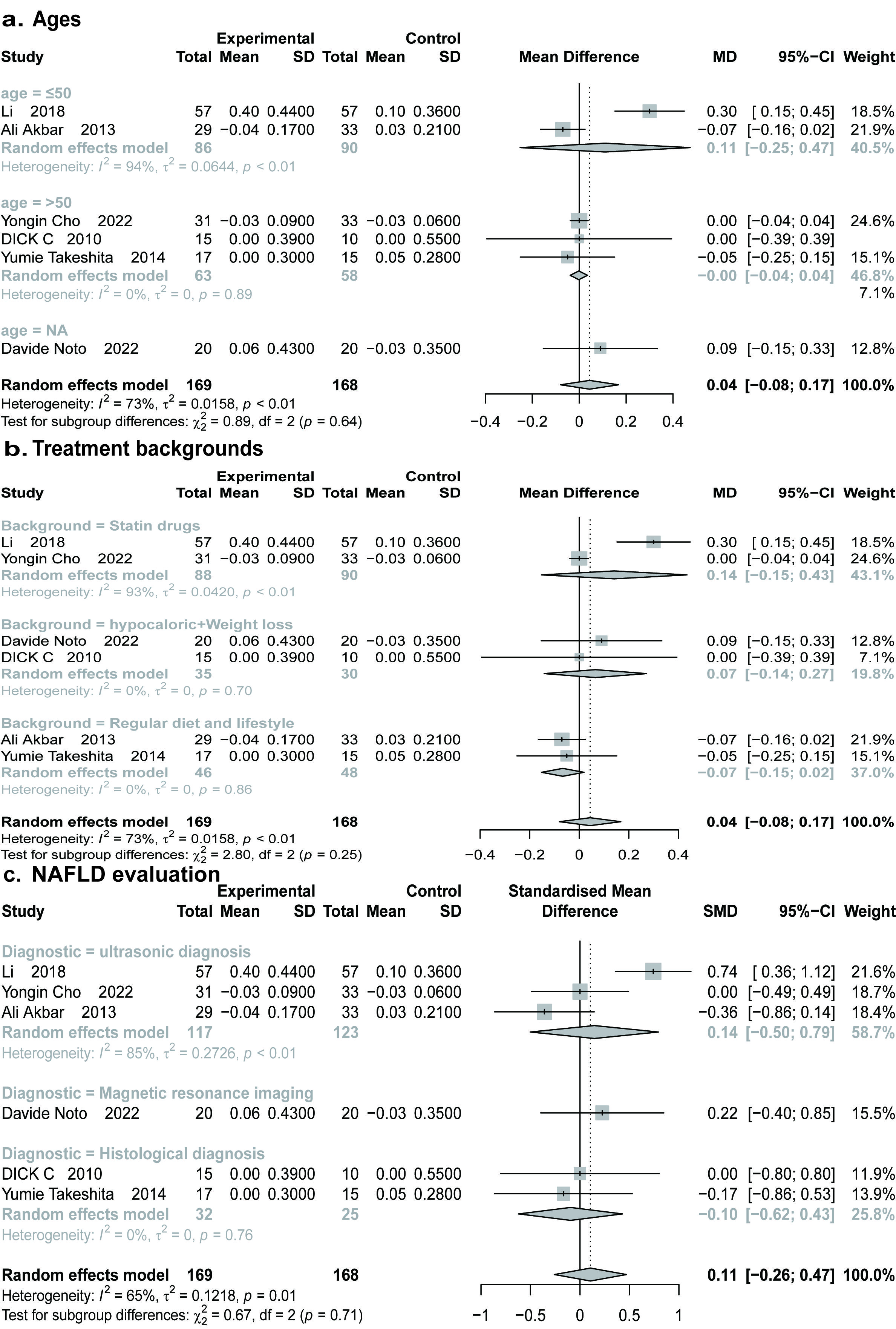


**Fig S7.** Meta-analysis of randomized controlled trials evaluating the impact of ezetimibe application on LDL-C levels in NAFLD patients across different control groups, treatment backgrounds (a), intervention periods (b), ages (c) and NAFLD evaluation(d).


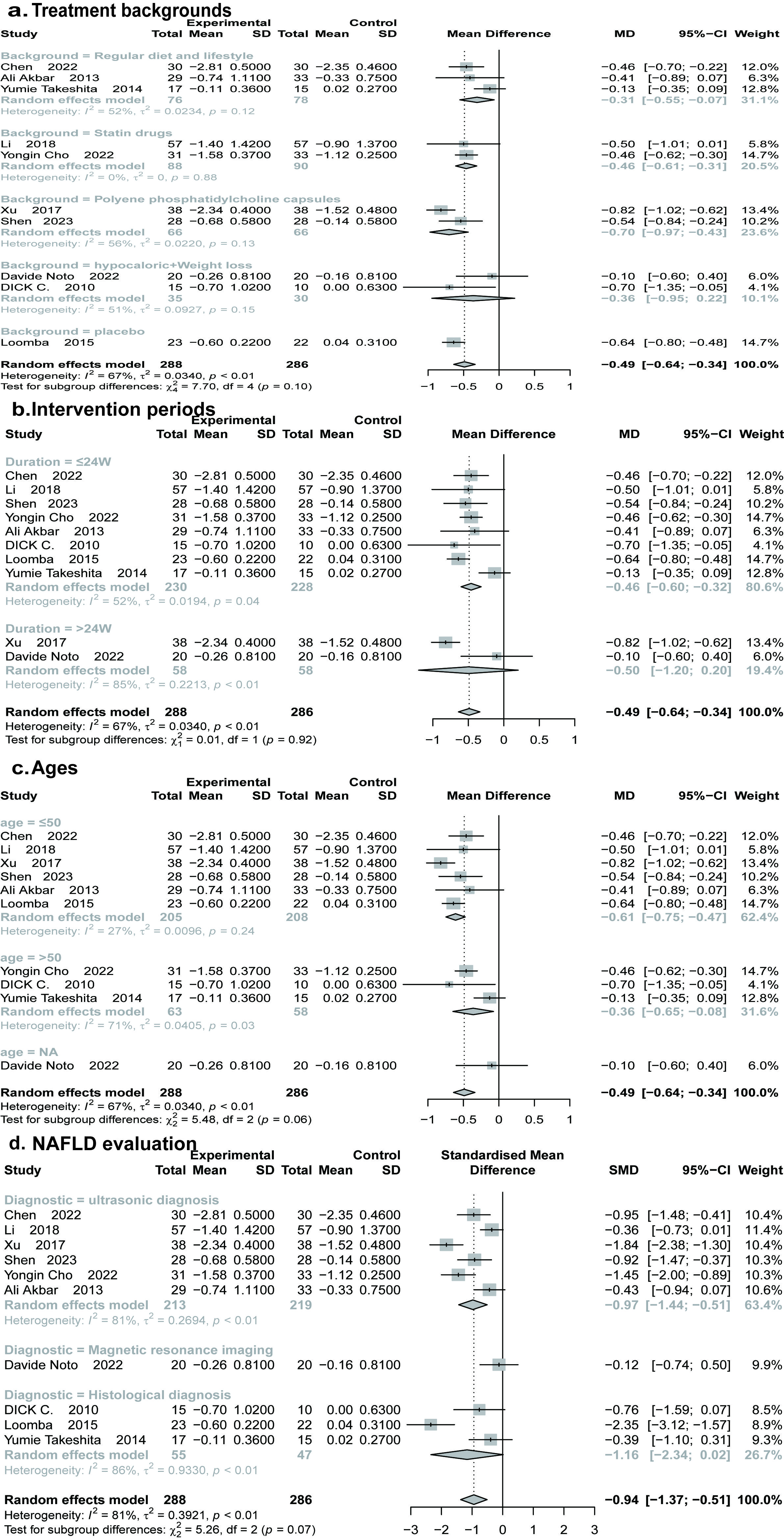


**Fig S8.** Meta-analysis of randomized controlled trials evaluating the impact of ezetimibe application on HOMA-IR levels in NAFLD patients across different control groups ages (a) and NAFLD evaluation(b).


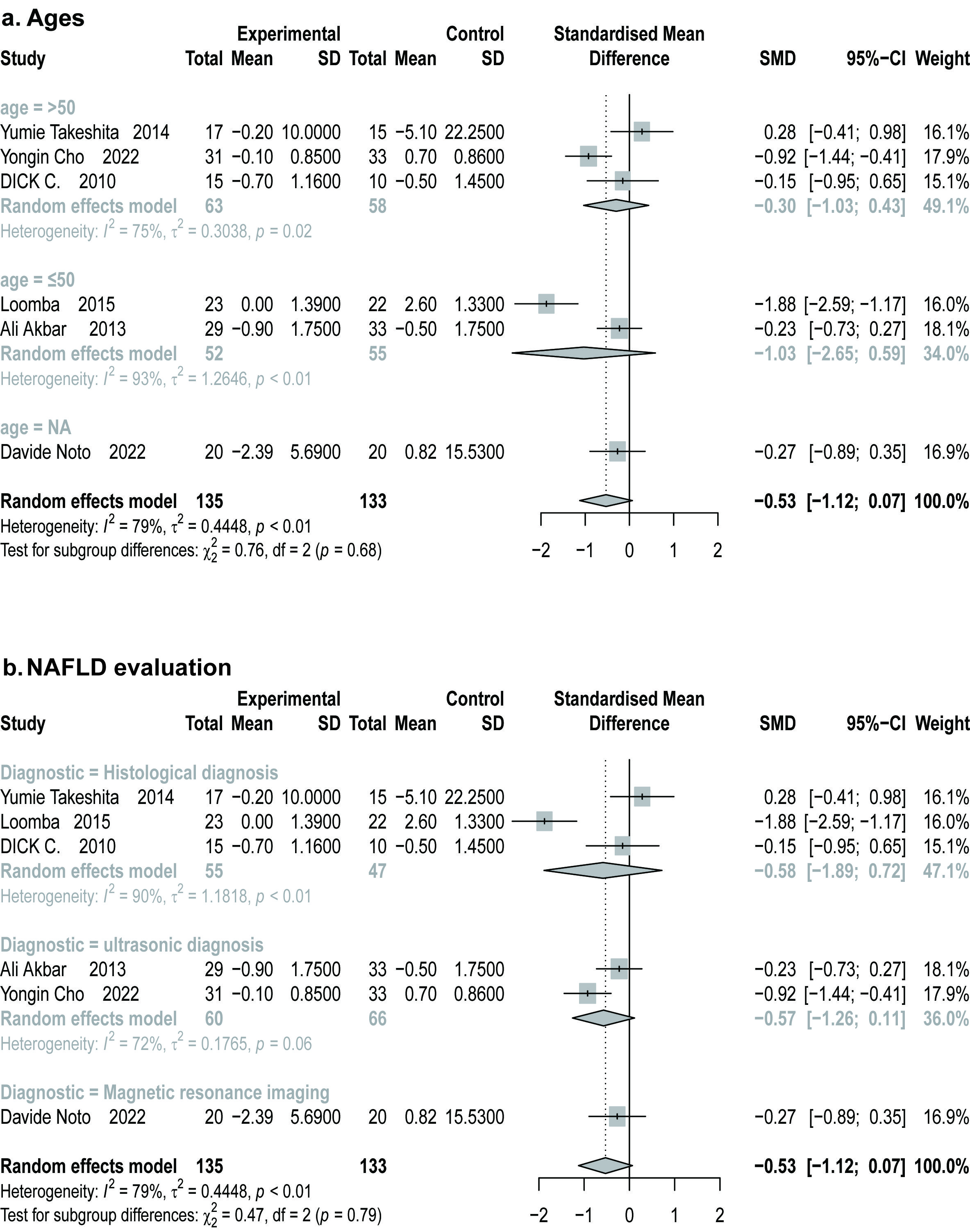

Supplement: Supplementary file 1 [file DataSheet1.doc]
